# Long-term response on letrozole for gastric cancer: A case report

**DOI:** 10.1097/MD.0000000000026146

**Published:** 2021-05-28

**Authors:** Yuuki Iida, Kumiko Hongo, Takanobu Onoda, Yusuke Kita, Yukio Ishihara, Naoki Takabayashi, Ryo Kobayashi, Ken Kuriki, Takeyuki Hiramatsu

**Affiliations:** a Department of Surgery, Yaizu City Hospital; b Department of Pathology, Yaizu City Hospital 1000 Dobara, Yaizu city, Shizuoka, Japan.

**Keywords:** case report, estrogen receptor, gastric cancer, hormone therapy, letrozole

## Abstract

**Rationale:**

Hormone therapies, particularly those targeting estrogen and its receptors, are a key treatment modality for patients with estrogen receptor (ER)-positive breast or ovarian cancer. Some gastric cancers (GCs) express ERs, and preclinical studies suggest the potential of estrogen-targeting hormone therapy on GC; however, the clinical relevance of this hormone therapy on GC treatment has not been well elucidated.

**Patient concerns:**

An 80-year-old female was admitted to our department with hypogastric pain and vomiting. Computed tomography demonstrated small bowel obstruction, and laparotomy after bowel decompression revealed peritoneal dissemination consisting of a poorly-differentiated adenocarcinoma. Intestinal bypass between the ileum and transverse colon was performed.

**Diagnoses:**

The tumor was ER- and mammaglobin-positive, indicating that it originated from a breast cancer. Diagnostic imaging revealed no evidence of breast cancer; however, right axillary ER- and mammaglobin-positive lymphadenopathy was found.

**Interventions:**

The patient received hormone therapy using letrozole based on a clinical diagnosis of occult breast cancer with peritoneal dissemination and right axillary lymph node metastasis.

**Outcomes:**

The patient remained disease free until 37 months but deceased at 53 months from the onset of disease. An autopsy revealed no tumor cells in the right breast tissue; however, there was a massive invasion of cancer cells in the stomach.

**Lessons:**

A patient with ER positive GC with peritoneal dissemination and right axillary lymph node metastasis presented remarkable response to letrozole. The long-term survival obtained using letrozole for a patient with GC with distant metastasis suggests the potential of estrogen targeting hormone therapies for GC.

## 1 Introduction

Currently, systemic therapies for unresectable locally advanced, recurrent or metastatic gastric cancer (GC) include chemotherapies and a limited variation of molecular targeted therapies.^[1]^ Preferred regimens for first-line therapy are a combination of fluoropyrimidines (e.g., fluorouracil or capecitabine) and platinum-based agents (e.g., cisplatin or oxaliplatin). ECF (epirubicin, cisplatin, and fluorouracil), DCF (docetaxel, cisplatin, and fluorouracil) and their modifications are also applicable for the first-line therapy.^[1]^ Preferred second-line therapy is a combination of taxanes (paclitaxel or nab-paclitaxel) and ramucirumab.^[1–3]^ Other chemotherapeutic agents, such as irinotecan, docetaxel, or trifluridine/tipiracil, are also candidates for second- or later-line treatment.^[1,4]^ Besides these chemotherapies, molecular targeted therapies have been recently applied in clinical practice; trastuzumab for HER2-positive GC, ramucirumab targeting VEGF2R used in second-line treatment with taxanes, and nivolumab, a monoclonal anti-programed death-1 antibody, known as immune checkpoint inhibitor.^[1,5]^ Despite these advances in chemotherapies and molecular targeted therapies for GC treatment, many of the clinical trials demonstrated median overall survival less than 18 months for unresectable locally advanced, recurrent, or metastatic GC; thus, alternative therapies need to be investigated to improve the prognosis of this disease.

Recently, many investigators have explored the potential of applying hormone therapy on GC, particularly targeting estrogen and estrogen receptor (ER).^[6–9]^ Hormone therapy targeting estrogen consists of an aromatase inhibitor, an ER inhibitor (tamoxifen), and a selective ER degrader (fulvestrant).^[10]^ Aromatase inhibitor, such as letrozole, exemestane, or anastrozole, are applied for the treatment of breast cancer in postmenopausal women. On the contrary, tamoxifen is mainly selected for premenopausal women with breast cancer.^[10]^ Although several reports have documented the effect of hormonal replacement therapy (HRT) or tamoxifen use on GC development,^[11–16]^ studies investigating the potential of estrogen targeting hormone therapy on GC treatment are currently very limited, and there are no recent reviews on this topic. Herein, we report a case of GC that presented with a long-term response on letrozole and reviewed recent progress in estrogen targeting hormone therapy for GC.

## 2 Case report

**Figure 1:**
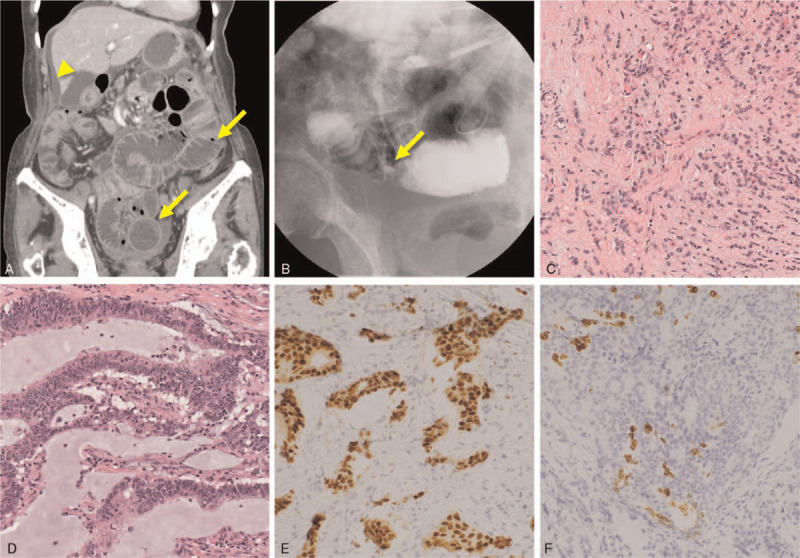
ER-positive poorly-differentiated adenocarcinoma from peritoneal dissemination. (A). Contrast-enhanced CT scan of the abdomen showing dilated small bowel (arrows) and ascites on the liver surface (arrowhead). (B). Gastrografin contrast radiography through long-tube showing a stenosed segment in the small bowel. Histopathological findings showing the proliferation of poorly-differentiated adenocarcinoma in the disseminated tissue (C, ×200) and proliferation of moderately-differentiated adenocarcinoma in the ascending colon cancer (D, ×200). Immunohistochemical examination showing positive results for ER (E, ×200) and mammaglobin (F, ×200) from the disseminated tissue.

An 80-year-old female, who underwent right hemicolectomy with lymph node dissection for ascending colon cancer one and half year ago (moderately-differentiated adenocarcinoma, Stage IIIa, UICC eighth edition), was admitted to our department with hypogastric pain and vomiting. The patient had slight anemia (Hb 10.3 g/dL) and high CA125 (902 U/mL), but CEA (3.1 ng/mL), CA19-9 (20.2 U/mL), CA72-4 (0.9 U/mL), and CA15-3 (11.4 U/mL) were within normal range. Computed tomography (CT) demonstrated small bowel obstruction and ascites in the pelvis and on the liver surface (Fig. 1A). After decompression of the small bowel with long-tube insertion, injection of contrast medium through the tube demonstrated a stenosed segment in the small bowel (Fig. 1B). The patient underwent laparotomy, which revealed peritoneal dissemination of a poorly differentiated adenocarcinoma diagnosed by intraoperative rapid diagnosis. For relieving the small bowel obstruction due to dissemination, intestinal bypass between the ileum and transverse colon was performed. The patient experienced no postoperative complication and started oral intake 4 days after the surgery.

**Figure 2:**
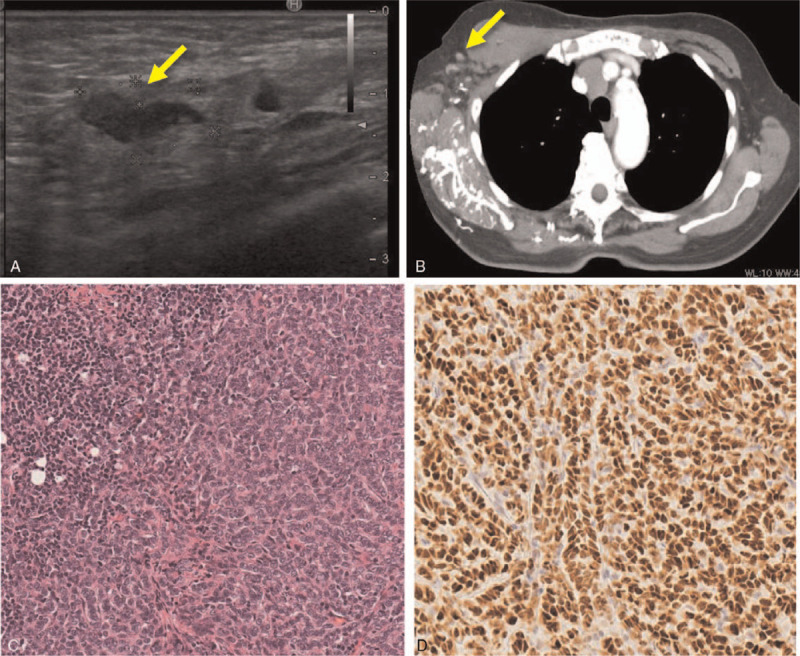
Proliferation of ER-positive poorly-differentiated adenocarcinoma in the right axilla. Ultrasonography (A) and CT (B) images showing lymphadenopathy in the right axilla. (C). Histopathological examination showing proliferation of the poorly-differentiated adenocarcinoma in the right axilla (×150). (D). Immunohistochemical examination showing positive result for ER (×150).

**Figure 3:**
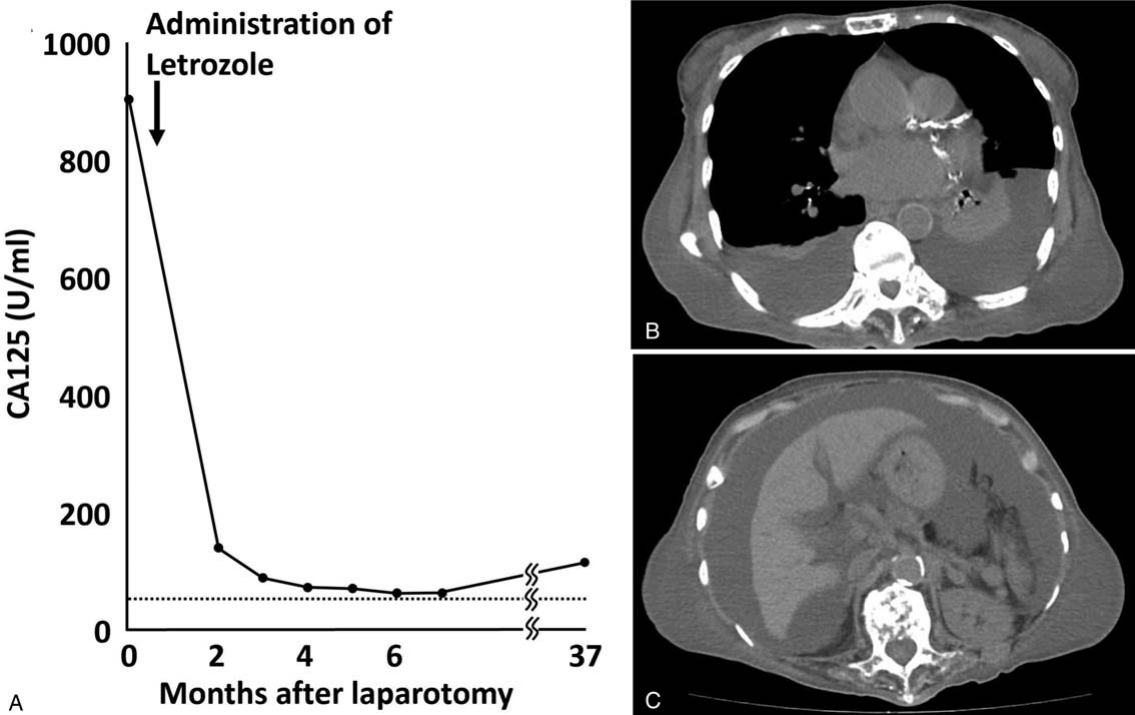
Clinical course with letrozole treatment. (A). Chronological change in serum CA125 level after laparotomy. CT scan showing pleural effusion (B) and ascites (C) at 48 months.

Histopathological examination confirmed proliferation of a poorly-differentiated adenocarcinoma in the resected specimen from the dissemination. Its feature was distinct from moderately-differentiated adenocarcinoma observed in the previous ascending colon cancer (Fig. 1C and 1D), suggesting the presence of an independent primary tumor. Immunohistochemical examination demonstrated positive results for cytokeratin (CK) 7, CK19, ER, progesterone receptor, and mammaglobin, whereas negative for CK20, GCDFP15, TTF1, or CDX-2 (Fig. 1E and 1F). The presence of ER, progesterone receptor, and mammaglobin expression indicated that the tumor originated from breast cancer. Ultrasonography, CT, and magnetic resonance imaging revealed lymphadenopathy in the right axilla (Fig. 2A and 2B); however, there was no evidence of breast cancer. The patient underwent surgical removal of the lymph node under local anesthesia, and histopathological and immunohistochemical examination demonstrated a similar pattern from the peritoneal dissemination (ER and mammaglobin-positive, Figures 2C and 2D). Based on these findings, we made a diagnosis of occult breast cancer with peritoneal dissemination and right axillary lymph node metastasis. Additionally, 18F-fluorodeoxyglucose positron emission tomography (PET)/CT, esophagogastroduodenoscopy, and colonoscopy revealed no evidence of other primary tumors. The patient and her family declined intensive chemotherapy for breast cancer to avoid severe adverse effects and decided to receive hormone therapy using letrozole for ER-positive breast cancer. Ascites diminished and CA125 rapidly decreased (Fig. 3A), and the patient remained free of disease until 37 months after the treatment without experiencing severe adverse effects. CT scan revealed ascites, pleural effusion, and multiple bone metastasis at 48 months (Fig. 3B and 3C), and she deceased from the cancer at 53 months.

**Figure 4:**
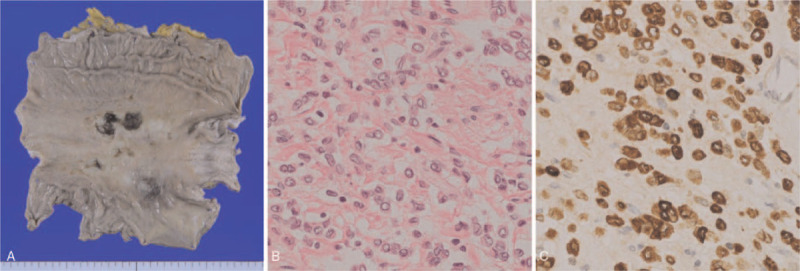
ER-positive gastric cancer specified by an autopsy. (A). Macroscopic image of the stomach obtained from an autopsy showing stiffness and thickening of the gastric wall. (B). Histopathological examination showing proliferation of poorly differentiated adenocarcinoma in the stomach (×400). (C). Immunohistochemical examination showing positive results for ER (×400).

An autopsy was performed to confirm the clinical diagnosis and treatment. The autopsy revealed no tumor cells in the right breast tissue; however, massive invasion and proliferation of poorly-differentiated adenocarcinoma was observed in the stomach (Fig. 4A and 4B), indicating that the tumor originated from GC. Interestingly, the tumor cells presented positive mammaglobin expression (Fig. 4C); however, no cells expressed ER, suggesting that the tumor cells acquired resistance during hormone therapy under drug pressure. The final diagnosis was ER-positive GC with peritoneal dissemination and right axillary lymph node metastasis; therefore, we concluded that GC presented remarkable response to letrozole.

## 3 Discussion

### 3.1 ER expression in GC

The prevalence of ER-positive GC varies between literatures and differs between ER subtypes, namely ERα and ERβ.^[17,18]^ Both ERs are members of a superfamily of nucleus receptors and exert their functions through a genomic pathway; estrogen binding promotes ER dimerization, the complex translocates into the nucleus, binds to estrogen response elements on the genomic DNA with transcriptional co-activators or co-repressors, and regulates DNA transcription of specific genes. There also exists a non-genomic pathway; ERs interact with other signaling molecules involved in MAPK or PI3K/Akt pathways.^[17,18]^ Interestingly, at sub-saturating hormone levels, ERβ functions as an inhibitor/competitor of ERα transcriptional activity, suggesting the relative expression level of the ER subtypes determine cellular responses to ER agonists and antagonists.^[17,19]^ Accordingly, the association between ER expression and clinicopathological features of GC differs between ERα and ERβ. There exist 3 isoforms for ERα, namely ERα66, ERα46, and ERα36, and their difference lies in their distinct transcriptional activation factors (AF-1 and AF-2). Of clinical relevance, ERα66 expression is associated with diffuse type GC, shorter DFS, or poor OS,^[8,17,20,21]^ whereas ERα36 expression is correlated with lymph node metastasis in clinical samples.^[22]^ Five different isoforms, ERβ1- ERβ5, have been identified for ERβ. Contrary to ERα, ERβ expression is associated with lower tumor stage, intestinal type, and free of recurrence.^[17,23]^ Another study demonstrated the absence of ERβ as an independent factor for poor OS, indicating the suppressive effect of ERβ on GC progression.^[21]^ These results suggest that ERα and ERβ have distinct effects on GC progression and that distinguishing ER subtype is essential. A recent meta-analysis also demonstrated that high ERα predicted poor OS and lower tumor differentiation, while high ERβ suggested favorable OS and higher tumor differentiation,^[24]^ further supporting the distinct function between ER subtypes in GC.

The prevalence of ER-positive GC ranges between 4.3% to 49.6% for ERα^[8,25,26]^ and 32% to 93.5% for ERβ.^[25–27]^ The positivity rates vary between studies, probably due to the difference in clinical background and evaluation (e.g., staining procedures and threshold for positive ER staining). As a considerable number of GC cases are ER positive, preclinical and clinical studies investigating the interaction between estrogen, ER, and GC potentially provides us additional strategies to overcome GC with metastasis.

### 3.2 Effect of HRT and tamoxifen use on GC development

In contrast to a limited number of investigations on hormone therapy in the treatment of GC, more reports have referred to the risk of GC development after HRT or tamoxifen use (an inhibitor of ER).^[11–16]^ Epidemiologic studies demonstrating male dominance of GC suggest a possible role of sex hormones on the oncologic risk of GC.^[28]^ A population-based study from Shanghai indicated that female hormones play a protective role in GC risk.^[12]^ Another population-based study from Sweden also demonstrated that the incidence of esophageal cancer and GC decreased with HRT.^[13]^ These results indicate the protective role of estrogen on GC risk. Conversely, tamoxifen use might accelerate GC progression or increase the GC risk based on population-based studies^[14,15]^; however, a recent meta-analysis demonstrated that there was no substantial GC risk with tamoxifen use in female patients.^[16]^ These studies refer to the preventive effect of HRT or potential risk of tamoxifen use on the development of GC; the effect of long-term exposure to estrogen or tamoxifen on the oncogenesis or progression of GC.

### 3.3 Potential of hormone therapy for GC treatment

Several preclinical studies using GC cell lines suggest the potential of applying estrogen-targeting hormone therapy on GC. One of the ER-targeting agents, tamoxifen, has an anti-proliferative effect on GC cell lines.^[9]^ Stimulation of GC cell lines with 17β-estradiol (E2) promoted proliferation, up-regulated ER-α36 mRNA expression, and repressed cell apoptosis. Conversely, tamoxifen treatment repressed proliferation, downregulated ER-α36 mRNA expression, and induced apoptosis, indicating the suppressive effect of tamoxifen on GC growth. Exemestane, an inhibitor of aromatase enzymatic function, also demonstrated a similar effect on GC cells.^[7]^ Aromatase is a key enzyme that catalyzes the conversion of androstenedione or testosterone to estradiol or estrone, and a high expression of aromatase was associated with poor overall survival in patients with GC. Exemestane inhibited aromatase and suppressed colony formation both in GC cell lines and a xenograft mouse model. Interestingly, the addition of 5-FU facilitated the suppressive effect of exemestane.^[7]^ Another agent, fulvestrant, an analog of E2 that downregulates and degrades ERα, also demonstrated anti-neoplastic efficacy on GC cells.^[8]^ E2 enhanced the proliferation of ER-positive GC cell lines, while the administration of fulvestrant repressed the proliferative effect of E2. Furthermore, fulvestrant presented synergistic anti-proliferative effect with paclitaxel.^[8]^ These results from preclinical studies provide a rationale for estrogen-targeting hormone therapy for GC treatment. Of clinical importance, the above-mentioned agents are already applied in clinical practice for patients with ER-positive breast cancer, thus these therapies are well characterized and managed for their adverse effects.^[10]^

Clinically, there is only 1 report demonstrating the clinical effect of hormone therapy on GC.^[6]^ They reported a case with ER-positive breast cancer treated with letrozole. The patient also suffered from independent primary GC with no ER expression and decided to initially start with letrozole treatment to regulate the breast cancer. Following the letrozole treatment, the patient underwent a staging laparoscopy followed by a subtotal D2 gastrectomy. Surprisingly, histopathological examination demonstrated no evidence of malignancy from the resected stomach and lymph nodes. Their report suggests the possibility of applying letrozole for GC treatment in clinical practice, although the mechanisms by which letrozole exerted its suppressive effect on ER-negative GC is unclear. In our case, metastatic GC showed positive ER expression, providing stronger rationale for the application of hormone therapy in GC. Patients with GC with peritoneal metastasis demonstrates poor OS for less than 18 months even with intensive surgery and chemotherapy.^[29,30]^ However, our case demonstrated remarkable response to hormone therapy and long-term survival for more than 50 months. To the best of our knowledge, we report the first case demonstrating long-term repression of ER positive GC using letrozole.

### 3.4 Applying hormone therapy on cancer of unknown primary with positive ER expression

The initial diagnosis in our case was occult breast cancer with peritoneal dissemination and right axillary lymph node metastasis; however, the origin of the tumor was GC at the final diagnosis. Immunohistochemical examination is indispensable to precisely specify the origin of the tumor for patients presenting a non-stereotypical clinical course as in this case or those with cancer of unknown primary (CUP).^[31]^ Breast cancer has multiple specific immunohistochemical markers, such as GATA3, mammaglobin, and GCDFP-15^[32–34]^; however, there are no specific markers strongly recommended or commonly used for GC, making it difficult to specify the origin in GC cases.^[31]^ Five percent of CUP cases are originating from GC based on autopsy,^[35]^ and they are potential candidates for hormone therapy when ER expression is positive. Currently, estrogen-targeting hormone therapy for ER-positive CUP is not recommended unless breast cancer is expected as an origin^[10,31]^; however, exploring GC specific markers as well as investigating estrogen-targeting hormone therapy for GC potentially improves the prognosis of CUP that are actually originated from GC.

## 4 Conclusions

We report a case of ER-positive GC with peritoneal dissemination and right axillary lymph node metastasis that displayed remarkable response on letrozole and long-term survival. Accumulation of GC case series treated with hormone therapy and further preclinical studies are encouraged for the wide application of hormone therapy on GC, and eventually, to facilitate clinical trials.

## Author contributions

**Conceptualization:** Yuuki Iida, Kumiko Hongo, Takanobu Onoda, Ryo Kobayashi.

**Data curation:** Yuuki Iida, Kumiko Hongo, Yusuke Kita, Naoki Takabayashi, Ken Kuriki.

**Supervision:** Yukio Ishihara, Ken Kuriki, Takeyuki Hiramatsu.

**Writing – original draft:** Yuuki Iida, Kumiko Hongo, Yusuke Kita, Yukio Ishihara, Naoki Takabayashi.

**Writing – review & editing:** Yuuki Iida, Kumiko Hongo, Takanobu Onoda, Yusuke Kita, Ryo Kobayashi, Ken Kuriki, Takeyuki Hiramatsu.
